# Birth preparedness and complication readiness among recently delivered women in chamwino district, central Tanzania: a cross sectional study

**DOI:** 10.1186/s12978-015-0041-8

**Published:** 2015-05-16

**Authors:** Deogratius Bintabara, Mohamed A. Mohamed, Janneth Mghamba, Peter Wasswa, Rose N.M Mpembeni

**Affiliations:** College of Health and Allied Sciences, University of Dodoma, P.O. Box 259, Dodoma, Tanzania; Ministry of Health and Social Welfare, Dar Es Salaam, Tanzania; African Field Epidemiology Network (AFENET), P.O. Box 12874, Kampala, Uganda; Muhimbili University of Health and Allied Sciences, P.O. Box 65015, Dar Es, Salaam, Tanzania

**Keywords:** Birth preparedness, Complication readiness, Obstetric danger signs, Chamwino, Tanzania

## Abstract

**Background:**

Unacceptably high maternal mortality rates remain a challenge in developing countries such as Tanzania. Birth Preparedness and Complication Readiness is among the key interventions that can reduce maternal mortality**.** Despite this, its status in Tanzania is not well documented. We assessed the practice and determinants of Birth preparedness and complication readiness among recently delivered women in Chamwino district, Central Tanzania.

**Methods:**

A community based cross-sectional study was conducted to women who delivered two years prior to survey in January 2014 at Chamwino district, Tanzania. Woman was considered as prepared for birth and its complication if she reported at least three of these; know expected date of delivery, saved money, identified a skilled birth attendant/health facility, mode of transport and Identified two compatible blood donors. Descriptive, bivariate and multivariable logistic regression analyses were performed at P value < 0.05 level of significance.

**Results:**

We interviewed 428 women whose median age (IQR) was 26.5 (22–33) years. About 249 (58.2 %) of the respondents were considered as prepared for birth and its complications. After controlling for confounding and clustering effect, significant determinants of birth preparedness and complication readiness were found to be maternal education (AOR = 2.26, 95 % CI; 1.39, 3.67), spouse employment (AOR = 2.18, 95 % CI; 1.46, 3.25), booking at ANC (AOR = 2.03, 95 % CI; 1.11, 3.72), Four or more antenatal visits, (AOR = 1.94, 95 % CI; 1.17, 3.21) and knowledge of key danger signs (AOR = 4.16, 95 % CI; 2.32, 7.45). Prepared for birth was found to be associated with institutional delivery (AOR = 2.45, 95 % CI; 1.12, 5.34).

**Conclusion:**

The proportion of women who prepared for birth and its complications were found to be low. District reproductive and child health coordinator should emphasis on early and frequent antenatal care visits, since they were among predictors of birth preparedness and complication readiness.

## Background

Maternal mortality is a major grobal concern [1]. sub-Saharan Africa has the highest maternal mortality ratio (MMR) averaging about 500 maternal deaths per 100,000 live births [2, 3]. Tanzania is among the developing countries with the highest MMR estimated to be 454 maternal deaths per 100,000 live births in 2010 [4]. Among the reasons for this high rate is inadequacy or lack of birth and emergency preparedness, which is a crucial component of globally recognized safe motherhood programs [5].

Birth preparedness and complication readiness (BPCR) can promote health care seeking behavior and utilization of appropriate heath care facilities and skilled personnel for delivery and hence reduce maternal death [6, 7]. This BPCR is the process of planning for normal birth and anticipating the actions needed in case complications arise. It promotes active preparation and decision making for delivery by pregnant women and their families [8]. Also it encourage women and their families to identify a skilled birth attendant; identify the location of the closest appropriate care facility; identify two compatible blood donors, save money for birth-related such as transportation to the health facility for obstetric emergency/delivery; identify of a person to take care of the family in case of the expectant mother’s absence; prepare important supplies for caring the newborn, being aware of the expected date of delivery and on the key obstetrics danger signs [9, 10].

BPCR strategy was adopted in Tanzania soon after implementation of focused antenatal care (FANC) in 2002 for the purpose of increasing health facility deliveries [10]. Through FANC women are equipped with health education and receive counseling on how to practice BPCR which may improve early decision making and care-seeking in case of delivery or obstetric emergency [10]. In this strategy government ensures that all health care providers in antenatal care (ANC) are well oriented with knowledge of counseling pregnant women on BPCR. Therefore it is expected that women who attend ANC receive counseling on BPCR and also on pregnancy, delivery and post-delivery danger signs [10]. Since implementation of BPCR through FANC, Tanzania Demographic Health Survey (TDHS) report of 2010 shows that, there is a slight increase in facility deliveries (47 % to 50 %) and decrease in home deliveries (53 % to 48 %) compared with the proportions observed in the 2004–05 TDHS [4, 11]. Lack of birth preparedness and insufficient preparation for quick action in case of an obstetric complications, contributes to delay in receiving skilled obstetric services [12]. Birth and emergency preparedness helps to ensure that women can reach skilled delivery care when labor begins and decreases the delays that occur when women experience obstetric complications [13]. Several studies have been conducted in sub-Saharan Africa found that women are not well prepared for birth and its complications, only 17 % from central Ethiopia [14], 22 % from north Ethiopia and 35 % in rural Uganda [15].

Twelve year post implementation of BPCR in Tanzania Still its practice and determinants have not been well studied both in urban and ruralsetting. Therefore we assessed practice and determinants of BPCR among recently delivery women in Chamwino district, central Tanzania. The findings of this study will provide important information regarding prevalence, practice and determinants of BPCR. They will also help the Ministry of health and social welfare, policy makers and public health specialists to design community based interventions which will encourage pregnant women and their families to be prepared for birth and its complications.

## Materials and methods

### Study area

The study was conducted in Chamwino district which is one of the the seven districts of Dodoma Region. The district had 63 functioning health facilities, including one district hospital, five public rural health centres, 55 government dispensaries, and two faith based dispensaries. Chamwino district had an estimated 77,429 women of reproductive age (15–49 years) in 2013, 24,242 live births in theprevious two years and a crude birth rate (CBR) of 38.5 births per 1000 population [16, 17].

### Study design and population

We conducted a cross-sectional in January 2014 that interviewed recently delivered women (RDW), defined as those women who delivered within two years prior to data collection regardless of newborn outcome.

### Sample size and sampling technique

We calculated the sample size using the cluster sampling formula adopted from United Nations guideline [18], which provided a sample size of 410 women based on the following assumption: proportions of pregnant women who prepared for birth and its complications were estimated at 86 % from Mpwapwa district Tanzania [19], level of significance at 95 %, Marginal error of 5 %, Design effect of 2 and by considering 10 % non-response rate.

A multi-stage cluster sampling technique was used. At each stage a sampling frame was developed and simple random sampling technique was employed. First, Chamwino district constitutes 32 wards. To determine the representative sample of Chamwino district, 1/8th of the wards were selected. Based on above calculation, four wards were selected randomly from all the 32 wards which constitute the district. From each selected ward, two villages were randomly chosen. Next, one hamlet was randomly chosen from each selected village. All households in each selected hamlet with RDW were visited in sequence and those who were eligible were enrolled. Where more than one RDW found in the same household, only one was randomly selected by using the lottery method. RDW of permanent resident of the Chamwino district, willing to participate and respond to the questionnaire were included in the study. Women who were not permanent resident, not willing to participate in the study, mentally disabled and severely ill were excluded.

### Measurements of variables

#### Socio demographic variables

Maternal age were grouped into “<20”, “20-29”, “30-39”, “>40”. Marital status were grouped into “single”, “ married/cohabiting” and “Widow/Separated/Divorced”. Later on grouped into “living with partner” and “not living with partner”. Education status grouped into “none”, “primary” and “secondary and above” leter on into “primary and above” and “no education”. Occupation was grouped into “Employed/Self-employed” and “Not employed”. Spouse’s education status was categorized into “none”, “primary” and “secondary and above” leter on into “primary and above” and “no education”. Spouse’s occupation status was categorized into “Employed/Self-employed” and “Not employed”. Monthly income of the mother in terms of cash were grouped into Tanzanian shillings “<50,000/=”, “50,000-100,000/=” and “>100,000/=”. Parity grouped into “1”, “2-4” and “≥5”.

#### ANC and reproductive health variables

Gestational age at booking categorized as “1st semester” and “other”. Number of ANC visit are grouped into “≥4 visits” and “<4 visits”. Receive counseling on birth preparedness (BP) was coded as “Yes” and “No”. Received counseling on complication readiness (CR) was coded as “Yes” and “No”.

Knowledge of the key obstetric danger signs during pregnancy, childbirth and postpartum: A woman was considered knowledgeable if she spontaneously mention a total of five danger sign in all three phases with at least one in each of phase. Phase 1: Danger signs during pregnancy (vaginal bleeding, swollen hands/face and blurred vision). Phase 2: Danger signs during labour/childbirth (severe vaginal bleeding, prolonged labour (>12hours), convulsion and retained placenta). Phase 3: Danger signs during postpartum (severe vaginal bleeding, foul-smelling vaginal discharge and high fever). This method of scoring has been previously used to assess women’s knowledge on obstetric danger signs [20].

The woman was assessed for the presence of the following five basic components of BPCR i) knew the expected date of delivery (EDD), ii) identified a skilled birth attendant or health facility for delivery/emergency, iii) identified mode of transport for delivery and obstetric emergency, iv) Saved money, v) Identified two compatible blood donors. A woman was considered as “prepared” for birth and its complication if she reported to follow at least three of five basic components of BPCR and the rest were considered as not “prepared”. This scoring has been previously used in studies which assessed women’s level of BPCR [5, 15].

Institution delivery: A woman was considered as having institution delivery if she was assisted by skilled birth attendant with midwifery skills (Physicians, Nurses, Midwives, and Health Officers) who can manage normal deliveries and diagnose, manage or refer obstetric complications.

### Data collection, management and analysis

Interviewer administered questionnaires were used during data collection due to the fact that 40 % of the population of Chamwino district are illiterate [16]. This questionnaire was adapted from a safe motherhood questionnaire developed by the Maternal Neonatal Program of JHPIEGO and modified to fit Tanzanian context. The expert translator translated it from English version to the local language (Swahili), and then another translator did a back translation to English to check for its original meaning. It was pretested in the neighboring district of Dodoma municipal. Five research assistants who have diploma in clinical medicine were trained for 3 days, participated in the pretesting and thereafter conducted the interviews under the supervision of principal investigator. All questionnaires were counter checked for completeness and consistency of the responses before leaving the field site. Those which were not filled properly, we returned back to the respective household and filled the information correctly.

Data was analyzed using STATA version 11.2. During descriptive analysis, continuous variables were summarized using mean and standard deviation while categorical variables were summarized using proportions and then presented in tables and graphs. Bivariate analysis was then done to test for associations between the dependent variable BPCR and other independent variables using Pearson’s chi square of Fischer’s exact test where appropriate. Then, all variables which showed association at bivariate analysis at (P value < 0.2) were fitted into the multiple logistic regression model by stepwise (forward selection) method to test for the association of each with the dependent variable at 95 % confidence level. Then final model obtained includes all variable which determine the BPCR. P-value and 95 % confidence interval (CI) for odds ratios (OR) were used to confirm the significance of the associations. P-value less than 0.05 was considered significantly different. In all our analyses we used the “svy” set command in Stata to adjust for clustering effect due to complex sampling.

### Ethical considerations

Ethical clearance was approved by Muhimbili University of Health and Allied Sciences (MUHAS) Research Ethics Committee. Permission was also obtained from Chamwino district executive director, wards executive officers, and village executive officers. A written informed consent has been obtained from all the respondents after being explained about all the relevant aspects of the study, including its aim, interview procedures, anticipated benefits and potential hazards. Also their right to refuse not to participate in the study any time they want was assured.

## Results

### Socio-demographic characteristics of study population

A total of 428 RDW were recruited and participated in the study making a response rate of 100 %. The median age (IQR) of the respondents was 26.5 [22–33] years. Of the respondents, majority 77.8 %, (333/428) were currently living with their partner (married or cohabiting). Among all respondents, 27.8 % (119/428) had not received any kind of formal education (Table 1).

**Table 1:** Socio-demographiccharacteristics of the respondents, Chamwino district, Central Tanzania, January, 2014 (N = 428)

Variable	Frequency	Percentage
Age years (Median 26.5, Range 16,50)		
<20	41	9.6
20-29	227	53.0
30-39	135	31.5
≥40	25	5.8
Marital status		
Single	70	16.4
Married/Cohabiting	333	77.8
Widow/Separated/Divorced	25	5.8
Educational status		
None	119	27.8
Primary	286	66.8
Secondary or above	23	5.3
Occupation		
Employed/Self-employed	74	17.3
Not employed	354	82.7
Spouse’s education status*		
None	53	15.9
Primary	254	76.3
Secondary or above education	26	7.8
Spouse’s occupation*		
Employed/Self-employed	52	15.6
Not employed/Peasant	281	84.4
Monthly income		
<50,000/=	381	89.0
50,000-100,000/=	40	9.3
>100,000/=	7	1.6
Parity		
1	108	25.2
2-4	208	48.6
≥5	112	26.2

*Could not add up to 428 because some of the respondents did not have spouse at the time of survey

### Utilization of ANC services

All of the respondents reported that they attended ANC during their last pregnancy. Majority of respondents 316 (73.8 %) had attended the minimum recommended number of four visits or more. Only a few respondents 74 (17.3 %) booked for ANC in the first trimester of their pregnancy. About 386 (90.2 %) and 363 (84.8 %) of respondents, reported to receive counseling on birth preparedness and on complication readiness respectively.

### Practices on Birth preparedness and complications readiness

Saved money was the most common element of BPCR reported by majority of respondents 360 (84.1 %). Most of the respondents 333 (77.8 %) had also identified either a skilled birth attendant or health facility for delivery/emergency. However, only 75 (17.5 %) of the respondents identified two potential blood donors who would donate blood in case of an obstetrics emergency (Table 2).

**Table 2:** Practices on birth preparedness and complications readiness, Chamwino district, Tanzania, January, 2014 (N = 428)

Variable	Frequency	Percentage*
Saved money	360	84.1
Identify a SBA or HF for delivery/emergency	333	77.8
Knowing expected date of delivery	208	48.6
Identify transport for delivery/obstetric emergency	146	34.1
Identify 2 blood donors	75	17.5

*Could not sum up to 100 % because multiple responses were possible

### Score on five basic components of BPCR

**Fig. 1:**
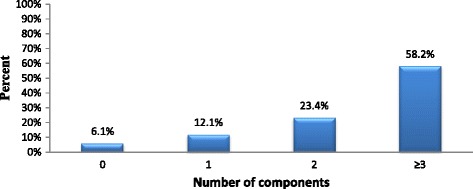
Number of basic components of birth preparedness and complication readiness reported (N = 428)

More than half 250 (58.4 %) of the respondents scored at least three of the five components of BPCR and were considered as prepared for birth and its complications while the rest were considered as not (Fig. 1).

### Knowledge of key danger signs during pregnancy, childbirth and postpartum

Overall 68.7 %, (294/428) of the respondents were not able to mention obstetric danger signs in any of three phases. Only 23.6 %, (101/428) of the respondents were able to mention at least a total of five key danger signs in all three phases and considered as knowledgeable on the key danger signs during pregnancy, childbirth/labour and postpartum (Table 3).

**Table 3:** Number of key danger signs during pregnancy, delivery and postpartum, Chamwino district, Central Tanzania, January, 2014 (N = 428)

Score on key danger sign	Frequency	Percentage
0	294	68.7
1-4	26	6.1
≥5	108	25.2
Total	428	100.0

### Determinants of BPCR

The baseline model in multiple logistic regression includes the following variables; BPCR as dependent variable and marital status, education, maternal occupation, spouse’s occupation, monthly income, parity, booking at ANC, knowledge on danger signs, counseling on BPCR as indepent variables. The final multiple logistics regression model showed that, the odds of birth BPCR were two times greater among women who received at least primary education when compared to those with no education (AOR = 2.26, 95 % CI; 1.39, 3.67). Occupation of the spouse was found to be significant determinant of BPCR, the odds of BPCR were two times greater among women whose spouse were employed, compared to women whose spouses were not employed at the time of the survey (AOR = 2.18, 95 % CI; 1.46, 3.25). Also, the odds of BPCR were two times greater among women who booked for ANC during the first trimester compared to those who booked after first trimester (AOR = 2.03, 95 % CI; 1.11, 3.72). Furthermore, the odds of BPCR were two times greater among women who attended at least four ANC visits compared to those with less than four visits (AOR = 1.94, 95 % CI; 1.17, 3.21) and finally, the odds of BPCR were four times greater among women who had knowledge of key danger signs than those who not had knowledge on key danger signs (AOR = 4.16, 95 % CI; 2.32, 7.45) (Table 4).

**Table 4:** Determinants of birth preparedness and complications readiness adjusted for confounding variables, Chamwino district, Central Tanzania, January 2014 (N = 428)

BPCR			COR (95 %:CI)	AOR(95 %:CI)
	Yes N(%)	No N(%)		
Marital union				
Living with partner	201 (80.7)	131 (73.2)	1.53 (0.66,3.55)	1.36 (0.79, 2.33)
Not living with partner	48 (19.3)	48 (26.8)	1.00	1.00
Educational status				
Primary and above	200 (80.3)	109 (60.9)	2.62 (1.51,4.55)	2.27 (1.07, 4.80)
No education	49 (19.7)	70 (39.1)	1.00	1.00
Occupation				
Employed	49 (19.7)	25 (14.0)	1.51 (0.98,2.34)	0.95 (0.35, 2.64)
Not employed	200 (80.3)	154 (86.0)	1.00	1.00
Spouse’s occupation*				
Employed	41 (20.3)	11 (8.4)	2.78 (1.69,4.56)	2.18 (1.46, 3.25)
Not employed	161 (79.7)	120 (91.6)	1.00	1.00
Monthly income				
≥50,000/=	36 (14.5)	11 (6.1)	2.58 (0.85,7.86)	1.68 (0.35, 8.09)
<50,000/=	213 (85.5)	168 (93.9)	1.00	1.00
Parity				
1	71 (28.5)	37 (20.7)	1.53 (0.79, 2.93)	1.99 (0.92, 4.30)
≥2	178 (71.5)	142 (79.3)	1.00	1.00
GA at booking				
1st trimester	53 (21.3)	21 (11.7)	2.04 (0.79,5.23)	2.52 (1.02, 6.18)
Other	196 (78.7)	158 (88.3)	1.00	1.00
No. of ANC visits				
≥4 visits	197 (79.1)	119 (66.5)	1.90 (1.27,2.84)	1.73 (1.02, 2.95)
<4 visits	52 (20.9)	60 (33.5)	1.00	1.00
Counseled on Birth Preparedness				
Yes	234 (94.0)	152 (84.9)	2.48 (1.35,4.55)	1.23 (0.61, 2.47)
No	15 (6.0)	27 (15.1)	1.00	1.00
Counseled on Complication Readiness				
Yes	223 (89.6)	140 (78.2)	2.29 (1.26,4.16)	1.96 (0.92, 4.17)
No	26 (10.4)	39 (21.8)	1.00	1.00
Knowledge on danger signs				
Yes	89 (35.7)	19 (10.6)	4.68 (2.38,9.23)	4.42 (1.94, 10.45)
No	160 (64.3)	160 (89.4)	1.00	1.00

*Could not add up to 428 because some of the respondents did not have spouse at the time of survey

### Influence of BPCR on Institutional delivery

**A**fter adjustment for other variables, odds of utilizing health facility for delivery was four times higher for women who had BPCR than for those who not had BPCR (AOR = 3.91, 95 % CI; 2.44,6.27) (Table 5).

**Table 5:** Influence of birth preparedness and complications readiness on institutional delivery, Chamwino district, Central Tanzania, January 2014

Institution delivery			*COR (95 %:CI)	**AOR (95 %:CI)
	Yes N(%)	No N(%)		
Having BPCR				
Yes	202 (69.2)	47 (34.6)	4.25 (2.76,6.55 )	3.91 (2.44,6.27)
No	90 (30.8)	89 (65.4)	1.00	1.00

* Include adjustment for clustering effect at village level by using svy command in Stata

** For adjusted odds ratio, it has been adjusted for age, maternal education level, maternal occupation, spouse occupation, distance to health facility, monthly income, marital status, parity, early date of booking, No. of ANC visits, counsel on BPCR and knowledge on danger signs, also for clustering effect at village level by using svy command in Stata

## Discussion

We assessed practice and determinants of BPCR among recently delivered women in Chamwino district of central part of Tanzania.

The prevalence of BPCR was estimated to be 58.2 %. This prevalence was found to be higher than that of studies done in South Ethiopia, Uganda and India [5, 15, 21] but low compared with study done in Mpwapwa district, Tanzania [19]. The difference of findings may be due to that our study participants were recently delivered women while in South Ethiopia were pregnant women and in Uganda were both delivered and pregnant women. Pregnant women may not able to report whether they identified/prepared for things that they have not yet needed. Also our study used (3/5) components of BPCR to consider woman as prepared for birth and its complication while study in India used (3/4) components.

A high proportion of the respondents reported that, they saved money as one of preparation for childbirth. This finding is similar from studies done in Uganda, Kenya, Burkina Faso and Nigeria [15, 19, 22–24]. This high proportion is due to the fact that women know that having money can help them in buying important supplies or pay for transportation in case of obstetrics emergency. Findings from the study show that only small proportion of the respondents identified the two compatible blood donors, our findings are comparable with other studies which were done at Nepal, Ethiopia and Nigeria [19, 24–26]. This may be due to thought that blood transfusion is considered to be a critical condition and few women think they will reach that condition especially if they have not been told to be anaemic during pregnancy.

Evaluating level of BPCR among the women can also be measured by assessing knowledge of obstetrics danger signs [9, 13]. Knowledge of obstetrics danger signs is an essential step in recognizing complications and enables one to take appropriate action to access emergency care [27]. In our findings on the knowledge of obstetrics danger signs was found to be very low. Similar finding were observed in studies done in North Ethiopia, Uganda and Kenya [15, 22, 25]. This low knowledge suggests inadequate awareness on danger signs in this district which may be due to the fact that, the women could not remember well the danger signs at the time study conducted and probably because of the high proportion of women who had not received formal education. Since the data from this study shows that more educated women were able to report more danger signs.

Women who had at least primary education were more likely to be prepared for birth and its complications compared to those who did not. Similar findings has been observed in the study conducted in Mpwapwa district Tanzania, rural Uganda, North Ethiopia and Indore City India [19, 21, 25, 28]. This might be due to the fact that educated women know the importance of planning for birth, adhere to counseling provided at ANC, and also have the capability of making decisions on issues related to their health.

Women whose spouses were employed were more likely to be prepared for birth and its complications compared to women whose spouses were not employed at the time of the survey’. Our findings are comparable with the studies conducted in Southern Ethiopia and Uganda [5, 28]. This might be due to the fact that living with an employed spouse meant a greater likelihood of having cash that can be used to prepare for birth and its complications.

Early and frequent antenatal care attendances are important to identify and alleviate risk factors in pregnancy and to encourage women to prepare for birth [29]. Findings from this study show that very few women booked for ANC during the first trimester and those who did so were more likely to be prepared for birth and its complications compared to those who booked after first trimester. In contrast, a study done in Nigeria found that those who booked late were more likely to be prepared for birth and its complications [24]. The difference in these findings may be due to the fact that in Tanzania counseling on BPCR is done on each ANC visit and repeated counseling among those who book early for ANC may lead to adhere to counseling. Also the study in Nigeria used a different study population who were pregnant women while our survey used RDW’s. Issue of recall bias was minimal in study of Nigeria since survey on pregnant women were more likely to remember compared to survey on women who delivered years ago.

Also we found that women who attended to ANC for t least four times were more likely to be prepared for birth and its complications compared to those who attended less than that. This suggests that attending many antenatal care services visits was an opportunity to inform pregnant women and help to plan for the important components of BPCR. In Tanzania focused antenatal care guideline, counseling on BPCR is required in all visits and so it is expected that women who attended four or more ANC visits received repeated counseling on how to prepare for birth. This findings has also been reported by previous studies [5, 21].

Knowledge of key danger signs is necessary for encouraging women and their families to seek health care in case of complications. In this study, women who had knowledge on obstetrics danger signs were more likely to be prepared for birth and its complication compared to those who did not have. This can be explained by the fact that knowing obstetrics danger signs may encourage women to be prepared for birth because they know that when any danger sign occur, they are likely to be attended if they are in hospital [15, 19]. Also, health education on danger signs which is provided to women during ANC emphases prompt use of health services upon seeing danger signs.

We observed that women who prepared for birth and its complications were four times more likely to deliver at health facility compared to those who did not. Our findings are in agreement with other studies [21, 26]. This is related with the fact that women who prepared for birth, are more likely to know where to go for childbirth and tend to know the importance of having safe delivery which is usually available at health facility.

The strength of the study is that, community based survey was employed and the large representative sample was obtained by using cluster sampling technique. Also we used the trained health personnel in data collection.

The limitation of this study were; Information on BPCR was based on self-reported information from study respondents since women did not have written documents on BPCR. Therefore, they were likely to have forgotten older events related to BPCR To minimize this women who had delivered more than two years at time of survey were excluded.

Subjects in the same cluster may have shared similar characteristics; this could have distorted our results by either neutralizing or overestimate prevalence or association. This was taken care of by adjusting for clustering during data analysis.

## Conclusion

This study revealed that, the proportion of women who prepared for birth and they were ready for any pregnant related complication was not satisfactory.

Significant predictors for BPCR were found to be; education level, gestational age at booking for ANC, number of ANC visits, spouse employment status and knowledge of key obstetric danger signs.

The Council Health Management team should encourage women in Chamwino district, Tanzania to book early for ANC and attend all the four minimum recommended visits for ANC as they were found to be among the predictors of BPCR. District education officer should put effort to empower women with education, since maternal education was found to be associated with BPCR. Also community-based education on importance of BPCR should be done to increase access to institutional delivery.
